# Relationship Between Nasal Septal Deviation Angles and Turbinates: A Computed Tomography Study

**DOI:** 10.7759/cureus.35253

**Published:** 2023-02-21

**Authors:** Pelin Zeynep Bekin Sarikaya, Nuray Bayar Muluk

**Affiliations:** 1 Department of Radiology, Kırıkkale University Faculty of Medicine Hospital, Kırıkkale, TUR; 2 Department of Otolaryngology, Kırıkkale University Faculty of Medicine Hospital, Kırıkkale, TUR

**Keywords:** paranasal ct, inferior turbinate, superior sd angle (ssda), sd curve angle (sdca), septal deviation (sd)

## Abstract

Background

This study aimed to evaluate inferior turbinate hypertrophy caused by nasal septum deviation, nasal septal deviation (SD) angles, and age differences with the help of paranasal computed tomography (CT) and to investigate the relationship between these parameters.

Methodology

The paranasal sinus CT images of 100 patients (50 males and 50 females) were retrieved from the hospital’s picture archiving and communication system. In this retrospective study, patients were examined in two groups. There were 50 patients aged >35 years in group 1 and 50 patients aged <35 years in group 2. The SD side was determined using a coronal image and was mentioned as the ipsilateral side. The contralateral side of the SD side was mentioned as the contralateral side. Additionally, the SD curve angle (SDCA), superior SD angle (SSDA), and diameters and mucosal thicknesses of the inferior turbinates were measured. Concomitant ipsilateral sinusitis and ipsilateral concha bullosa (in the middle concha) were also noted as present or absent.

Results

In our study, the SDCA values of the ≥35-year age group were significantly higher than those of the <35-year age group (p < 0.05). Furthermore, the SSDA values of the ≥35-year age group were significantly lower than those of the <35-year age group (p < 0.05). In each of the age groups, ipsilateral inferior turbinate mucosal thickness and ipsilateral inferior turbinate diameter values were significantly higher than those of the contralateral sides (p < 0.05). Ipsilateral concha bullosa was present in 30.0% of the <35-year age group and 18.0% of the ≥35-year age group. Ipsilateral sinusitis was present in 34.0% of the <35-year age group and 52.0% of the ≥35-year age group.

Conclusions

SD and inferior turbinate hypertrophies should be evaluated together and measured with paranasal CT to provide more efficient nasal aeration. Studies with larger patient series are needed to elucidate the etiology.

## Introduction

Septal deviation (SD) and inferior turbinate pathologies are the most common causes of nasal obstruction [1]. The hypertrophy of the inferior turbinate toward the concave side of the SD is referred to as compensatory hypertrophy [2]. The most common non-infectious causes of inferior turbinate hypertrophy appear to be long-standing nasal SD or rhinitis [3].

Although there are many studies in the literature on this subject, its etiology and causes have not yet been fully clarified. Inferior turbinate hypertrophy, the cause of which cannot be understood and which cannot be fully treated, also reduces the chance of success in nasal SD surgeries [4].

The objective of this study was to evaluate inferior turbinate hypertrophy caused by nasal septum deviation (NSD), nasal SD angles, and age differences with the help of paranasal computed tomography (CT) and to investigate the relationship between these parameters.

## Materials and methods

This retrospective study was conducted at Kırıkkale University, Faculty of Medicine, Radiology and Otorhinolaryngology Departments according to the principles of the Declaration of Helsinki. Paranasal sinus CT (PNSCT) scans were retrieved from the digital database of the Radiology Department, Kırıkkale University, Faculty of Medicine. Ethics committee approval was obtained from Kırıkkale University Non-invasive Research Ethics Committee (date: 29.06.2022, number: 2022.06.27). There was no need to obtain informed consent because the data were evaluated retrospectively.

Study subjects

In this retrospective study, the PNSCT images of 100 patients (50 males and 50 females) were retrieved from the hospital picture archiving and communication system between August 2022 and January 2021. Patients were examined in two groups, with 50 patients aged >35 years in group 1 and 50 patients aged <35 years in group 2. The mean age of the males was 36.30 ± 12.68 years (range: 20-63). The mean age of the females was 39.0 ± 16.15 years (range: 18-77). According to the age distribution of our patients, we identified the young group as those younger than 35 years, wherein body development continues partially, osteoporosis is not observed, collagen production is active, and exposure to trauma is higher. A history of major trauma, S-shaped deviations, a sinonasal tumor or infection, nasal polyposis, or sinonasal surgical history were the exclusion criteria in this study. The inclusion criteria included patients older than 18 years who underwent a paranasal CT scan without meeting any exclusion criteria.

PNSCT imaging and analysis

All scans were obtained using routine PNSCT imaging in the supine position and the head positioning of hyperextension. Contrast or sedation was not used for the procedures. The images were acquired using a 64-slice CT (Brilliance 64, Philips Medical System, Best, the Netherlands). All scans were obtained using the following parameters: tube voltage = 120 kV, effective mAs = 350, slice thickness = 1.00 mm, the field of view (FOV) = 180 mm, and image matrix = 768 × 768. The images were transferred to a workstation, and the raw data were reconstructed using bone algorithms. After scanning, the coronal, axial, and sagittal images were reconstructed with a slice thickness of 1.00 mm. The coronal plane was often preferred. All patients included in this study were evaluated by the same radiology expert (PZBS) on a high-resolution monitor.

Measurements

The coronal plane was often preferred for measurements. The SD side was determined using a coronal image and was mentioned as the ipsilateral side. The contralateral side of the SD side was mentioned as the contralateral side.

**Figure 1:**
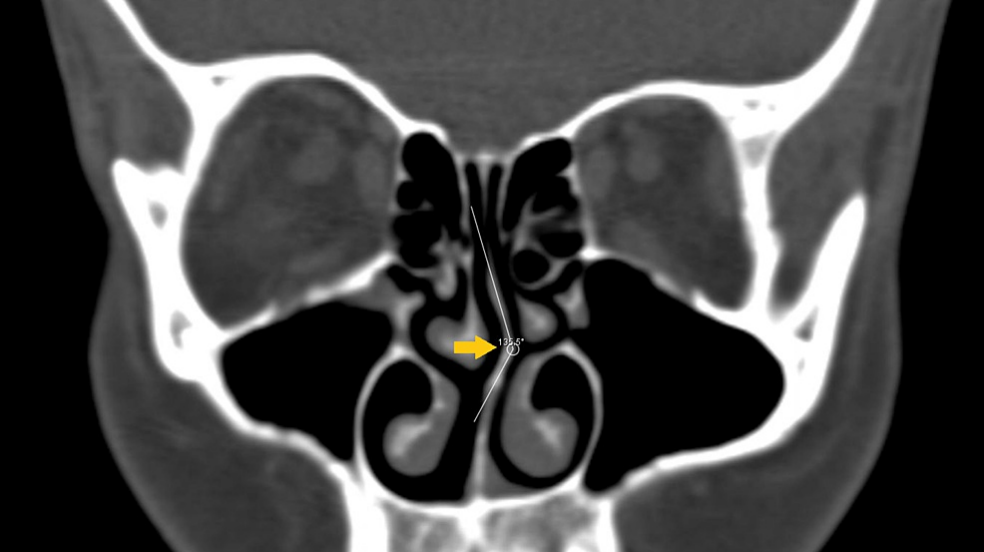
Septal deviation curve angle. On the coronal paranasal bone image, the septal deviation curve angle measurement is shown.

**Figure 2:**
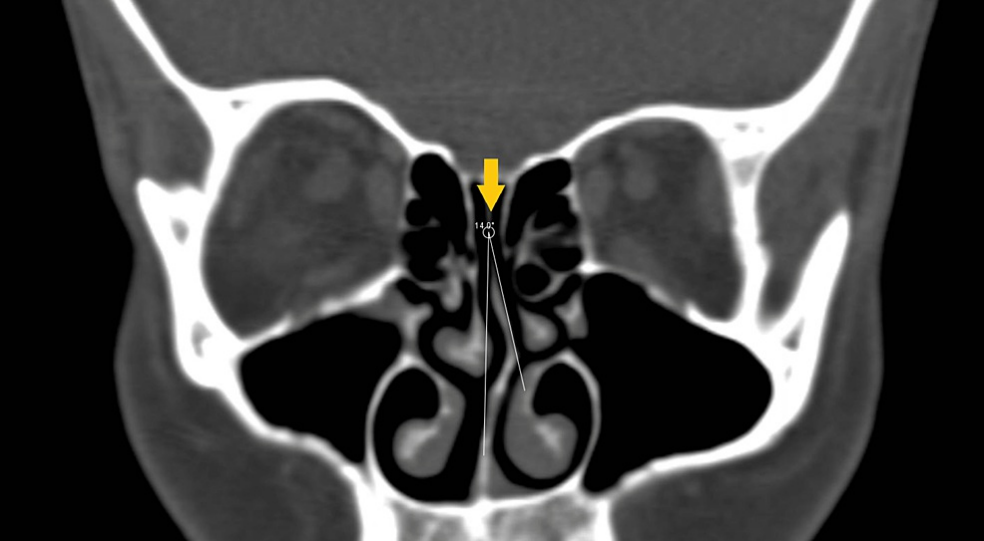
Superior septal deviation angle. On the coronal paranasal bone image, a superior septal deviation angle measurement is shown.

**Figure 3:**
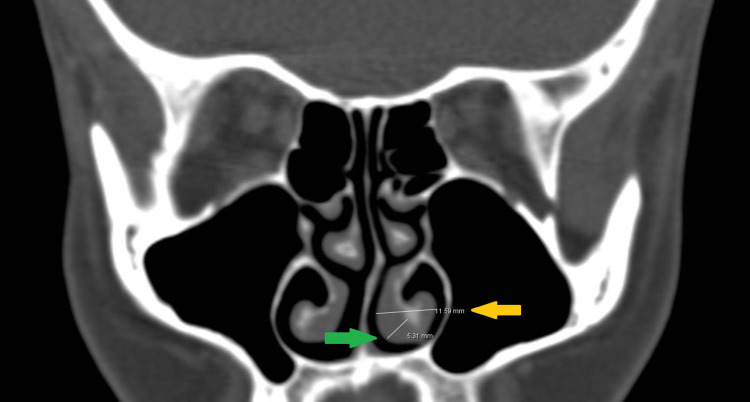
Ipsilateral inferior turbinate mucosal thickness and diameter. On the coronal paranasal image, ipsilateral inferior turbinate mucosal thickness (green arrow) and diameter (yellow arrow) measurements are shown in millimeters.

The SD angle was measured as the SD curve angle (SDCA). We took the highest point of the deviated septum as the edge and drew an obtuse angle from this edge point with converging septum edges (Figure 1). The nasal deviation angle was measured using coronal CT images as the angle between the most deviated point of the septum and the midline as the superior SD angle (SSDA). The line from the crista galli to the palatum was defined as the midline [5,6] (Figure 2). Furthermore, the diameters and mucosal thicknesses of the inferior turbinates were measured in millimeters by choosing the thickest section on the ipsilateral side (Figure 3); the contralateral sides were measured in the same way. Concomitant ipsilateral sinusitis and ipsilateral concha bullosa (in the middle concha) were also noted as present or absent.

Statistical analysis

SPSS version 21.0 (IBM Corp., Armonk, NY, USA) was used for statistical analysis. Independent-samples t-test, paired-samples t-test, chi-square test, Pearson correlation test, and Spearman’s correlation rho efficient test were used. A p-value <0.05 was considered statistically significant.

## Results

There were 50 male patients and 50 female patients. There were no significant differences between the ages of the males (mean age = 36.30 ± 12.68 years) and females (mean age = 39.0 ± 16.15 years) (p > 0.05). In all groups, 49.0% of the SDs were right-sided and 51.0% of the SDs were left-sided.

SDCA values of the ≥35-year age group (mean = 136.73 ± 18.63 degrees) were significantly higher than those of the <35-year age group (mean = 125.09 ± 21.53 degrees) (p < 0.05) (Table 1). SSDA values of the ≥35-year age group (mean = 22.53 ± 7.83 degrees) were significantly lower than those of the <35-year age group (mean = 18.61 ± 7.47 degrees) (p < 0.05) (Table 1). There were no significant differences between ipsilateral and contralateral inferior turbinate diameters in the <35-year and ≥35-year age groups (p > 0.05). In each of the age groups, ipsilateral inferior turbinate diameters were significantly higher than those of the contralateral sides (p < 0.05) (Table 1). There were no significant differences between ipsilateral and contralateral inferior turbinate mucosal thickness values in the <35-year and ≥35-year age groups (p > 0.05). In each of the age groups, ipsilateral inferior turbinate mucosal thickness values were significantly higher than those of the contralateral sides (p < 0.05) (Table 1). Ipsilateral sinusitis was present in 34.0% of the <35-year age group and 52.0% of the ≥35-year age group (p > 0.05) (Table 1). Ipsilateral concha bullosa was present in 30.0% of the <35-year age group and 18.0% of the ≥35-year age group (p > 0.05) (Table 1).

**Table 1:** Measurement results according to the age groups (<35 years vs. ≥35 years).

			Group 1 (<35 years) (n = 50)					Group 2 (≥35 years) (n = 50)				P-value
			Mean	Median			SD	Mean	Median		SD	
Age (years)			25.54	25.00			4.62	49.76	51.00		10.25	0.000
Measurement results												
Septal deviation curve angle			125.09		127.90		21.53	136.73	138.80		18.63	0.005
Superior septal deviation angle			22.53		21.65		7.83	18.61	18.05		7.47	0.012
Inferior turbinate diameter	Ipsilateral (septal deviation side)		11.71		11.55		2.78	11.39	11.05		2.68	0.552
	Contralateral		10.38		10.10		2.43	10.04	9.90		2.62	0.512
	P-value		0.003					0.002				
Inferior turbinate mucosal thickness	Ipsilateral (septal deviation side)		4.69		4.58		1.53	4.67	4.40		1.47	0.949
	Contralateral		3.99		3.60		1.49	3.95	3.75		1.32	0.888
	P-value**		0.001					0.001				
			n			%		n		%		P-value
Ipsilateral sinusitis		Absent	33			66.0		24		48.0		p = 0.069; χ^2^ = 3.305
		Present	17			34.0		26		52.0		
Ipsilateral concha bullosa		Absent	35			70.0		41		82.0		p = 0.160; χ^2^ = 1.974
		Present	15			30.0		9		18.0		

There were no significant differences between the SDCA values of the males (mean = 128.72 ± 20.14 degrees) and females (mean = 133.09 ± 21.55 degrees) (p > 0.05) (Table 2). There were no significant differences between the SSDA values of the males (mean = 21.72 ± 7.48 degrees) and females (mean = 19.41 ± 8.14 degrees) (p > 0.05) (Table 2). There were no significant differences between the ipsilateral inferior turbinate diameters of males and females (p > 0.05). However contralateral inferior turbinate diameters of males (10.72 ± 2.82 mm) were significantly higher than those of the females (9.70 ± 2.08 mm) (p < 0.05). In each of the gender groups separately, ipsilateral inferior turbinate diameters were significantly higher than those of the contralateral sides (p < 0.05) (Table 2). Ipsilateral and contralateral inferior turbinate mucosal thickness values of males were significantly higher than those of females (p < 0.05). In each of the gender groups separately, ipsilateral inferior turbinate mucosal thickness values were significantly higher than those of the contralateral sides (p < 0.05) (Table 2). Ipsilateral sinusitis was present in 56.0% of males and 30.0% of females (p < 0.05) (Table 2). Ipsilateral concha bullosa was present in 24.0% of males and 24.0% of females (p > 0.05) (Table 2).

**Table 2:** Measurement results according to the gender groups

		Group 1 (males) (n = 50)				Group 2 (females) (n = 50)				P-value
		Mean	Median		SD	Mean	Median		SD	
Age (years)		36.30	34.50		12.68	39.00	34.50		16.15	0.355
Measurement results										
Septal deviation curve angle		128.72	134.20		20.14	133.09	135.95		21.55	0.297
Superior septal deviation angle		21.72	21.10		7.48	19.41	18.40		8.14	0.143
Inferior turbinate diameter	Ipsilateral (septal deviation side)	11.90	11.72		2.83	11.20	10.87		2.59	0.205
	Contralateral	10.72	10.55		2.82	9.70	9.80		2.08	0.044
	P-value	0.013				0.000				
Inferior turbinate mucosal thickness	Ipsilateral (septal deviation side)	5.04	5.10		1.63	4.32	4.21		1.26	0.015
	Contralateral	4.46	4.54		1.58	3.48	3.40		1.00	0.000
	P-value	0.013				0.000				
		n		%		n		%		P-value
Ipsilateral sinusitis	Absent	22		44.0		35		70.0		p = 0.009; χ^2^ = 6.895
	Present	28		56.0		15		30.0		
Ipsilateral concha bullosa	Absent	38		76.0		38		76.0		p = 1.000; χ^2^ = 0.000
	Present	12		24.0		12		24.0		

There was a negative correlation between SDCA and SSDA values (p = 0.05, r = -0.555) (Table 3). There were positive correlations between ipsilateral and contralateral inferior turbinate diameters and ipsilateral and contralateral inferior turbinate mucosa thickness values (p < 0.05) (Table 3). In right-sided SDs, SDCA values were higher (p < 0.05) (Table 3). In males, ipsilateral and contralateral inferior turbinate mucosal thickness values and the presence of sinusitis values were higher than those in females (p < 0.05) (Table 3). In older SD patients and ≥35-year-old SD patients, SDCA values were higher and SSDA values were lower than in younger patients with SD (p < 0.05) (Table 3).

**Table 3:** Correlation test results in all septal deviation patients (n = 100).

			Septal deviation curve angle (SDCA)	Superior septal deviation angle (SSDA)	Inferior turbinate diameter		Inferior turbinate mucosal thickness		Presence of ipsilateral sinusitis (Code 0: Absent, Code 1: Present)	Presence of ipsilateral concha bullosa (Code 0: Absent, Code 1: Present)
					Ipsilateral (septal deviation side)	Contralateral	Ipsilateral (septal deviation side)	Contralateral		
SDCA		r		-0.555	0.085	-0.157	0.055	-0.147	0.104	-0.142
		P		0.000	0.399	0.119	0.586	0.146	0.305	0.159
SSDA		r	-0.555		-0.076	0.119	-0.040	0.079	-0.105	0.072
		P	0.000		0.454	0.239	0.693	0.436	0.297	0.478
	Ipsilateral (septal deviation side)	r	0.085	-0.076		0.367	0.721	0.332	-0.020	-0.046
Inferior turbinate diameter		P	0.399	0.454		0.000	0.000	0.001	0.847	0.651
	Contralateral	r	-0.157	0.119	0.367		0.201	0.754	0.036	0.076
		P	0.119	0.239	0.000		0.045	0.000	0.722	0.453
Inferior turbinate mucosal thickness	Ipsilateral (septal deviation side)	r	0.055	-0.040	0.721	0.201		0.516	0.013	-0.034
		P	0.586	0.693	0.000	0.045		0.000	0.901	0.736
	Contralateral	R	-0.147	0.079	0.332	0.754	0.516		0.026	-0.006
		P	0.146	0.436	0.001	0.000	0.000		0.795	0.955
Septal deviation side (Code 1: Right, Code 2: Left)		r	0.372	-0.138	0.033	-0.159	-0.010	-0.144	-0.118	-0.105
		P	0.000	0.170	0.748	0.115	0.924	0.154	0.241	0.299
Gender (Code 1: Male, Code 2: Female)		r	0.097	-0.165	-0.134	-0.176	-0.212	-0.315	-0.263	0.000
		P	0.335	0.100	0.182	0.079	0.034	0.001	0.008	1.000
Age		r	0.271	-0.292	-0.108	-0.092	-0.044	-0.006	0.166	-0.129
		P	0.006	0.003	0.283	0.365	0.667	0.952	0.098	0.202
Age group (Code 1: <35 years, Code 2: ≥35 years)		r	0.290	-0.261	-0.062	-0.072	-0.009	-0.011	0.182	-0.140
		P	0.003	0.009	0.540	0.478	0.932	0.916	0.070	0.163
Presence of ipsilateral sinusitis (Code 0: Absent, Code 1: Present)		r	0.104	-0.105	-0.020	0.036	0.013	0.026		0.079
		P	0.305	0.297	0.847	0.722	0.901	0.795		0.432
Presence of ipsilateral concha bullosa (Code 0: Absent, Code 1: Present)		R	-0.142	0.072	-0.046	0.076	-0.034	-0.006	0.079	
		P	0.159	0.478	0.651	0.453	0.736	0.955	0.432	

## Discussion

NSD is a common variation with a variable incidence of 22-80% [6-9]. Septal surgery is one of the most common otolaryngological surgeries [10]. According to the literature, NSD has been associated with sinusitis, breathing difficulties, loss of sense of smell, apnea, recurrent sneezing, and epistaxis [11]. Quality of life is affected in patients with nasal obstruction due to tumors, and complications may occur after surgery [12]. For this reason, the diagnosis and surgical planning of this region should be optimized regardless of the disease. Diagnosis and treatment are important as they are associated with many clinical conditions. PNSCT is the most commonly used method for diagnosis. There is no standard grading for NSD in paranasal CT sections. Although there are various classifications of NSD in the literature, none of them are used alone in daily routine practice in ENT polyclinics. In addition, the components of the septal deviation that cause nasal obstruction and the most appropriate criteria for measurement have not yet been determined [13]. SSDA measurement is most frequently used in daily patient evaluations and NSD studies. We could not find any comparative study on SDCA in the literature. We found a negative correlation between SDCA and SSDA values in our study. As the SDCA narrows, the deviation of the nasal septum from the midline increases. Hence, the SDCA can also be used in daily practice together with SSDA.

In our study, the SDCA values of the ≥35-year age group were significantly higher than those of the <35-year age group. Furthermore, the SSDA values of the ≥35-year age group were significantly lower than those of the <35-year age group. The increase in NSD with increasing age can be explained by trauma and genetics [14,15]. According to some studies, the formation of NSD stops after the age of 49 as bone growth stops and exposure to trauma decreases [8]. Studies that include the correlation of pathology related to the loss of elasticity of the nasal septum and the change in the angle of deviation as age progresses are needed.

Inferior turbinates are an important part of nasal breathing. Environmental, allergic, and inflammatory agents influence the mucosa of turbinates reversibly. Inferior turbinate size changes can be observed permanently in some chronic conditions such as allergic rhinitis or NSD [16]. According to the literature, NSD frequently causes compensatory contralateral inferior turbinate hypertrophy [17,18]. However, in our study, in each of the age groups, ipsilateral inferior turbinate mucosal thickness and ipsilateral inferior turbinate diameter values were significantly higher than those of the contralateral sides. This different result may be due to mucosal irritation caused by the sharp part of the nasal deviation angle to the ipsilateral inferior turbinate. Moreover, the diurnal rhythm of erectile inferior turbinates may cause this result. Another reason may be the direction chosen by patients in their sleeping habits and applying asymmetric pressure to the turbinates. In addition, the fact that complex deviations were not included in the study may have affected this result. This result may be important as it may change the surgical approach. A surgery may fail due to inadequate treatment of the inferior turbinate after septoplasty operations. Therefore, the inferior turbinate should be considered during septoplasty planning [19,20]. After septal surgery, a hypertonic seawater solution (2.3% NaCl) was used by Lascaris et al. [21].

The pneumatization of the concha is termed concha bullosa. According to some studies, the incidence of concha bullosa varies due to the position of the septum [22,23]. The study by Stallman et al. found that the incidence of concha bullosa increased in the presence of NSD [24]. In the study of Yiğit et al., the overall incidence of concha bullosa was 31.52%, and it increased to 45.34% in patients with NSD [25]. We found that ipsilateral concha bullosa was present in 30.0% of the <35-year age group and 18.0% of the ≥35-year age group.

According to some studies in the literature, the incidence of maxillary sinusitis increases as NSD impairs nasal aeration and changes maxillary sinus volumes [26,27]. In our study, ipsilateral sinusitis was present in 34.0% of the <35-year age group and 52.0% of the ≥35-year age group. For our data on this subject to make sense, it is necessary to add it to our study with a control group.

There are some limitations of this study. One of them is the small number of patients. Large case series are needed for better results. Moreover, follow-up imaging is required so that compensatory changes during the day do not affect the study. Another limitation is the inability to correlate the etiological factors of the patients and the pathologies of the nasal septa. By including these factors, more useful results can be obtained. Another limitation is that complex and multiple NSDs were not included in the study. Studies on these complex NSDs may yield different results.

## Conclusions

NSD is quite common in daily practice and hospitalizations are required for surgical treatment. SD and inferior turbinate hypertrophies should be evaluated together and measured with paranasal CT to provide more efficient nasal aeration. In this way, we can increase treatment success and avoid repeat surgeries.
